# Viral Hepatitis Induces Hepatocellular Cancer: What Can We Learn from Epidemiology Comparing Iran and Worldwide Findings?

**DOI:** 10.5812/hepatmon.7879

**Published:** 2012-10-30

**Authors:** Elisabeth Smolle, Evelyn Zöhrer, Kira Bettermann, Johannes Haybaeck

**Affiliations:** 1 Institute of Pathology, Medical University Graz, Graz, Austria

**Keywords:** Viral Hepatitis, Carcinoma, Hepatocellular, Iran

## Abstract

**Context:**

Several risk factors play the role in the development of hepatocellular carcinoma (HCC) from which chronic hepatitis B and C infections are the most important ones. DNA integration of hepatitis viruses alters the function of critical genes promoting malignant transformation of virus-infected liver cells.

**Evidence Acquisition:**

There are remarkable geographic differences in prevalence of chronic viral hepatitis and incidence of HCC. Middle Eastern countries are characterized by a moderate to high prevalence rate of chronic viral hepatitis in the population. This review discusses about epidemiologic findings of hepatitis B and C infections, and HCC, as well as focuses on Middle East countries, particularly Iran. We provide an overview about risk factors, prevention and treatment, and bring up the role of HCC induced by chronic viral hepatitis.

**Results:**

Vaccination against hepatitis B virus (HBV) in the early childhood is highly effective to lower infection rates, substantially. For hepatitis C, adequate hygiene when dealing with human blood and screening programs for blood donors can mainly reduce infection rates. As HCC is strongly associated with chronic viral hepatitis, prevention against the infection is crucial for preventing against HCC too.

**Conclusions:**

Although prevention and treatment of chronic hepatitis B and C have improved within the last decades even in high-risk countries, effective and sustainable reduction of these infections still needs more actions.

## 1. Context

### 1.1. Chronic Viral Hepatitis

Several risk factors were found to play a role in the development of hepatocellular carcinoma (HCC), and chronic hepatitis B and C infections are reported as the most important underlying diseases. DNA integration of hepatitis viruses alters function of genes, which promote malignant transformation of virus-infected hepatocytes. Biochemical findings may include elevation of serum aminotransferases and mild elevation of alkaline phosphatase (1). If patients remain undiagnosed, they manifest symptoms of cirrhosis and chronic liver failure in the end stage such as more extreme fatigue and jaundice, anorexia, weight loss, or weight gain due to ascites (2). The incubation times in hepatitis B, C and D are different. For hepatitis B, it is variable and can range from 30 to 180 days. Incubation time in hepatitis C is similar, ranging from 15 to 180 days. If the first infection with hepatitis D occurs simultaneously with hepatitis B infection, the incubation time is quite short within three to seven weeks. Acute forms of hepatitis usually have much shorter incubation time and the first infection is more severe compared to chronic viral hepatitis.

### 1.2. Pathogenesis of Viral Hepatitis

It is the complex interaction of virus and the patient’s immune response that determines outcome of the disease (3). However, direct cytotoxicity of hepatotropic viruses does not play a significant role in hepatic injury, whereas host immunological responses do (2) The virus itself can cause mutations in infected cells leading to an escape of both humoral and cell-mediated immune responses (4, 5). Once the infection occurs, humoral antiviral immunity helps clearance of viruses from the body, but the intrahepatic process is rarely mediated by these antibodies. Cytotoxic T-lymphocyte (CTL) response together with CD4+ T-helper cells is probably the most important mechanism for elimination of intrahepatic viruses (2). CTLs induce death of infected hepatocytes by specific recognition of host and viral antigens on the surface of infected cells vascular injury (e.g. thrombosis) plays a role in the establishment of cirrhosis (4).

### 1.3. Multiple Chronic Viral Infections

Co-infection with hepatitis B, C, and D viruses are not uncommon since all of these viruses spread parentally. Several clinical reports on viral co-infections show that the clinical course is not significantly altered compared to a single viral infection (6). There are no particular histopathologic features that specifically reveal co-infections. On the immunohistochemical level, however, there are specific features pointing out to this. For instance, expressions of hepatitis B surface and core antigens (HBsAg and HBcAg) are suppressed by simultaneous HCV infection (2). Thus, in patients who are serologically positive for HBsAg but negative in tissue staining, clinical suspicion to co-infection with HCV should be raised (7). As human immunodeficiency virus (HIV) also spreads parenterally, co-infection of hepatitis and HIV is often observed. In the setting of HIV, HBV infection becomes persistent and leads to the establishment of chronic HBV-associated hepatitis. Furthermore, re-activation of HBV happens more frequently due to concomitant HIV infection. HIV might worsen the clinical course of concomitant chronic hepatitis C. Generally, HCV infection in HIV-infected patients has become a more important issue because new anti-retroviral therapies (8).

### 1.4. Individual Types of Chronic Viral Hepatitis (Hepatitis B)

**Figure 1:**
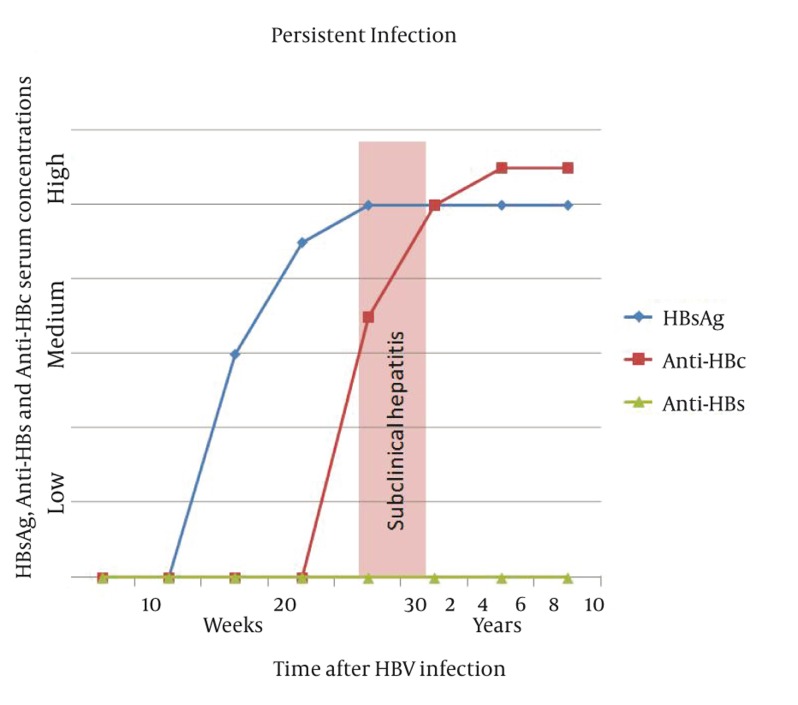
Serum Concentrations of HBsAg, Anti-HBs and Anti-HBc in Self- Limited HBV Infection

**Figure 2:**
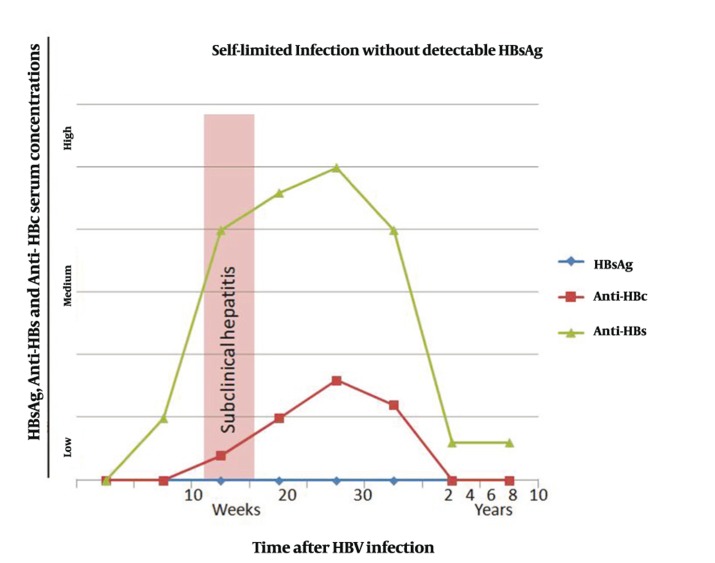
Serum Concentrations of Hepatitis B Surface Antigen (HBsAg), Anti-HBs and Anti-HBc in Persistent HBV Infection (HBc = hepatitis B core protein)

It is estimated that up to 350 million people are infected with HBV worldwide (2). Regions with a particularly high HBV incidence and prevalence are China, Southeast Asia and Sub-Saharan Africa (2). In these areas up to 15% of the population is infected (2). Viral replication takes place after the virus has entered the hepatocyte. During this process, viral DNA enters the nucleus and produces viral messenger RNA which serves as a template for viral DNA synthesis and is packaged with viral DNA-polymerase (reverse transcriptase) into a viral capsid composed of HBcAg (2). Within the capsid, double stranded viral DNA is produced and consecutively the core particles are assembled into complete virions with HBs Ag and cell membrane envelopes (2). HBV is a blood-borne virus and mostly spreads parenterally (2).Needle sharing in intravenous drug abusers is mainly responsible for new infection with HBV today. Previously, blood transfusion recipients were also at high risk of being infected with HBV, but rigorous screening of blood donors has nearly eliminated post-transfusion HBV infections. As HBV is found also in saliva and semen, spreading via kissing or sexual contact is possible. Vertical HBV transmission rarely occurs via a diaplacentar route, but frequently during the delivery. In children of industrialized countries, horizontal spread is very common which will presumably be changed in the next few decades with establishment of vaccination programs (9). HBV infection can be self-limited or persistent; the latter case leads to chronic hepatitis. In acute hepatitis, hepatitis B core and surface antigens (HBcAg and HBsAg) first appear in the serum around 8 weeks later (2). After HBsAg disappears from the patient’s serum, it usually takes several weeks for appearance of HBs antibodies (HBsAb). In self-limited HBV infection, however, HBsAg may never be detectable in the serum (Figure 1). HBs antibodies can persist in the patient’s serum for the entire life making the patient immune against a new HBV infection. In chronic HBV infection, HBsAg appears in the serum in a similar time course but there is no development of HBs antibodies (Figure 2). HBsAg persists for the patient’s lifetime. A distinctive feature is used to distinguish chronic hepatitis B from other forms of chronic hepatitis, and termed as ‘ground-glass hepatocyte’ (2).The term is used because of fine, granular cytoplasmic inclusions consisting of endoplasmic reticulum that is loaded by HBsAg (2). The cytoplasmic inclusion is usually surrounded by a clear halo which pushes the nucleus towards the cell margin (2).

### 1.5. Hepatitis B in Iran

The Islamic Republic of Iran is one of the largest countries in population with a low infection rate of HBV. After having realized the HBV National Vaccination Program for all neonates in 1993 and the vaccination of high risk groups, the prevalence of the virus decreased dramatically (10). According to the report of World Health Organization (WHO) in 2001 and Centers for Disease Control and Prevention (CDC) in 2005, prevalence of chronic hepatitis B infection in Iran ranges between 2-7% (11).

### 1.6. Risk Factors for HBV Infection Transmission in Iran

Possibilities for passing on HBV include unsafe sexual contact, blood transfusions, use of contaminated needles, and vertical transmission from mother to her neonate. To find transmission routes for hepatitis disease in Iran, a study was conducted in 2005 on 500 patients with chronic hepatitis B and 434 healthy volunteers and evaluated certain jobs and lifestyle characteristics as possible risk factors. The results revealed that age, marital status, sexual activity, transfusion history, imprisoned drug abusers, and particular job holders - like barber or policeman - influences the possibility to contract hepatitis B virus infection (Table 1) (12). In Iran, sexual habits are not similar to Western countries due to despite of differences in the religion and laws. Primarily, sexual activity starts averagely at higher age compared to Western civilization. Hepatitis B virus is mostly transmitted by sexual contacts. Interestingly, women more likely transmit the virus than men (13). Polygamy plays an important role as a risk factor for transmitting HBV, as well. Based on the Iranian culture polygamy is allowed for men. Women are prohibited to have extramarital sexual contacts resulting in the fact that in general, men play the notable role in HBV transmission (12). Intravenous drug addicts who share their needling with others are at high risk to transmit HBV. Some studies revealed that more than a quarter of intravenous drug users in prison (8.4% of all prisoners) in southern Iran were HBV carriers (14). The percutaneous route is a common way of transmission including piercing, tattooing, and special treatment with needles like acupuncture. Vertical transmission of HBV from infected mothers to neonates possibly occurs at three different periods: during pregnancy, at birth, and after delivery. In Western countries, the neonatal HBV infection transmitted from mother’s accounts for less than 10%. In 1979, a published study from Farzadegan et al. revealed that more than 50% of HBV infected individuals had mothers who were also positive for tender no longer necessary hepatitis B surface antigen (HBsAg) (15). It was suggested that more than half of Iranians affected by HBV received the virus perinatally, which highlighted this way as the most frequent route of transmission in Iran. However, since the beginning of the National Vaccination Program in 1993 the infection rate of neonates decreased significantly.

**Table 1:** Estimations of Risk Factors for HBV Infection in Iran

Risk Factors	Percentage (Range)
**Sex, Male**	2.4 (1.8-3.2)
**Maritus status, Married**	2.1 (1.4-3.2)
**Extramarital Sex**	6.5 (1.4-30.0)
**Intravenous Drug Abuse**	5.5 (1.2-25.3)
**High Risk Job**	2.4 (1.5-4.0)

## 2. Evidence Acquisitions

People working in healthcare areas were also classified as high risk group for viral transmission. Various types of surgery, dentistry, gynecology, cardiology, and gastroenterology procedures gynecological, cardiological and gastroenterological procedures pose the threat on infection. Additional routine vaccination programs for prisoners, under therapy drug addicts, and staffs of health-care centers may be efficient steps for further prevention.

### 2.1. Genotypes

Nine main genotypes of HBV, termed from A to I, have been described which show more than 8% variations in their DNA sequence (16). With respect to each other furthermore, these genotypes are divided into subtypes, which differ in nucleotide arrangement ranging from 4% to 8%. A number of studies investigating HBV positive individuals from different parts of the country proved by genome sequencing that genotype D1 are the predominant genotype in Iran (10, 17). The genome samples from Iranians were compared to other populations with predominantly genotype D in the eastern countries including Pakistan, Uzbekistan, Turkey, Egypt, and India. The closest resemblance was observed between HBV of Iranians, Pakistanis, and Turks. Genome sequencing of the virus also determined the main serotype demonstrating that ayw2 with 95.1% is the most common serotype in Iran, followed by ayw3 and ayw4 with 4.9%. The presences of special amino acids at certain position on the DNA of HBsAg (Arg122, Lys160 and Pro/Thr/Leu127) are defining these types of the hepatitis B virus (18). Different genotypes may also influence disease progression in various ways; however, these reports are controversial. Further investigation on HBV genomic variability may be a crucial goal for monitoring disease progression and designing specific therapeutic drugs for individual patients.

### 2.2. Mutations

Frequent mutations of certain genes in HBV infected patients were described which might influence not only the clinical outcome of the disease but also the response to therapy. HBV surface (S), precore (PC), and basal core promoter (BCP) genes are often mutated in individuals affected by this special virus (19) The S gene region codes for the surface antigen (HBsAg). Mutations occurring in the immunologic ‘a-determinant region’ allow the virus to evade the host’s immune system (19). Changes in S protein may also provide the escape of surface-antigen neutralization, hepatitis B immunoglobulin therapy, and distort the results of diagnostic serological tests. Mutations in the precore and basal core promoter may play an important role in the progression of liver disease. The most frequent one on the PC gene is G1896A mutation (20). The rate of HBV precore mutants in Iranian patients was reported to be between 55.6% in 2012 (17). PC G1896A mutants prevent the expression of fully intact HBe Ag protein by inserting a stop codon within the messenger RNA. As a result, HBV patients with a PC G1896A mutation lack the presence of HBe Ag. In 2012, Mohebbi et al. confirmed the influence of precore mutant G1896A by detecting this mutation with an increased frequency in patients affected by liver cirrhosis (77.2%) than in negative carriers (38.1%) (17). Another study investigated the influence of double mutation of precore and basal core promoter. It was shown that the presence of an A1762T/G1764A mutation of PC and BCP gene is linked with to an advanced, more aggressive form of liver disease (20).

### 2.3. Epidemiology of HBV in Iran

In Iran, the prevalence of HBV is reaching from 2 to 7% according to the WHO report in 2001 and CDC report in 2005 (11). In the 1980s the percentage of Iranians affected by chronic HBV accounted for 3%, reaching from a prevalence rate of 1.7% in the province of Fars to 5% in Sistan va Balouchestan. It was estimated that at that time one million out of 35 million Iranians were HBs Ag positive and about half of them were suffering from liver cirrhosis. The implementation of the national vaccination program, the clarification about hepatitis risk factors, and the vaccination of high risk groups led to a significantly drop of viral hepatitis infection in the Iranian population during the last decade. Whereas the number of HBV carriers declined, the average age of infected individuals increased. Due to effective mass vaccination of neonates, the infection rate in children of 2-14 years old of age decreased from 1.3% to 0.8% (P < 0.05) (21).

### 2.4. Geographic Distribution

Approximately 1.5 million Iranian people are infected with hepatitis B virus. Statistically, 15% to 40% of them will develop liver cirrhosis, and ultimately HCC without further treatment (22). HBV infection rates are varying in the provinces of the Islamic Republic of Iran (Table 2). The northeastern region of the country shows the most cases affected by HBV, whereas the prevalence in central and western Iran is significantly lower. The highest prevalence rate was seen in the province Golestan with 6.3% (23). Heterogeneous prevalence of HBV according to their geographical location should be investigated more carefully to clarify different predisposing factors to improve the efficiency of prevention.

**Table 2:** HBV Infection Prevalence in Different Iranian Provinces in the General Iranian Population

Province	Region	Percentage of HBV Infection Prevalence
**E.Azarbaijan (26-28)**	Nothwest	1.3
**Fars (37)**	South	1.7
**Golestan (38)**	North	6.3
**Hamedan (29)**	West	2.3
**Hormozgan (30)**	South	2.4
**Isfahan (31)**	Central	1.3
**Sistan-Balouchestan (36)**	Southeast	5.0
**Tehran (32-35)**	Central	2.2

### 2.5. Hepatitis C

HCV was identified first in 1989. It is assumed that about 3% of the world’s population is currently infected with HCV which adds up to more than 170 million chronic virus carriers (34). The virus mostly spreads parenterally, so there is a high HCV prevalence in intravenous drug abusers. In the 1950s and 1960s, recipients of blood transfusions were at high risk for HCV infection, too, but today careful virus screening in blood donors has almost eliminated post-transfusion infections. Other ways of viral spreading such as needle-stick injuries in healthcare jobs, tattoos, and piercings or intranasal cocaine use have also been reported (35). Sexual HCV transmission can occur but is rare and is usually associated with a dysfunctional immune system. Maternal-infant transmission is also rare (36). When HCV is acquired, patients are rarely symptomatic in first place, though it has been reported that fulminant hepatitis C can also occur (37). About 90% of acutely infected individuals move to chronic infection and consecutive liver injury (38). In the chronic state of hepatitis C, 25% of all patients will develop cirrhosis over time (38). Additionally, the risk for development of HCC is significantly increased in patients with chronic HCV infection (39). Histopathological findings in chronic hepatitis C include dense monocyte infiltration, interface hepatitis, and portal inflammation. There are microscopic features that are rather characteristic for chronic hepatitis C such as prominent lymphoid aggregates in the portal tract and destruction of bile ducts (2). It was shown that a strong proliferation of CD4+ T-cells and a distinct cytokine response to the infection are associated with viral clearance in the acute phase (40).Considering the paucity of lymphocytes in tissue sections, the primary damage is mainly virus-mediated rather than caused by the patient’s immune system (41).

### 2.6. Genotype

The WHO reported the presence of more than 21 million HCV carriers in the Middle Eastern countries. Studies concerning the genotype demonstrated that genotype 4 is prevalent in most of the Arabian countries, whereas genotype 1a and 1b predominates in non-Arabian countries (42). In Iran genotype 1a is predominant for HCV, followed by genotype 3a and 1b, in addition to mixed genotypes (43). Subtype 1a was the most frequent one in southern Iran making up to 70%, while type 3a was more common in north-western regions of the country (83%). In Tehran, hemodialysis patients showed a predominance of genotype 4. An accurate genotyping of the hepatitis C virus may improve treatment strategies and help designing both effective vaccines and specific therapeutic drugs for individual patients.

### 2.7. Epidemiology and Transmission

In Iran, there is no overall estimate of HCV infection rate. There are studies referred to different provinces and potential high-risk groups. The prevalence of HCV in Iran is influenced by different factors like geographical location, mass immigration, and importation of drugs from eastern bordering countries. In Iran, prevalence of infection varies throughout the country. Some studies reported prevalences of 15.6% in Fars, 44.3% in Kerman (44), 29.6% in Zahedan (45) 59.1% (36) in Hamadan, 71.3% in Gilan to 76.7% in north-west of Iran, representing an overall prevalence rate of almost 50%. Routes of transmission are mainly the exposure to infected blood or blood products, contaminated medical instruments, intravenous drug abuse, and organ transplantation. Iatrogenic transmission of HCV by blood transfusions and blood products such as coagulation factors in hemophiliac patients is still a common route. There is evidence supporting the theory that prisoners belong to a high-risk group for the prevalence of HCV. Syringe sharing is a more frequent procedure among intravenous drug users inside the prisons than that outside (42). This route of transmission is strongly and independently associated with both hepatitis C and HIV infections (42) The offer of sterile injection equipment may present an easier opportunity to stem the problem of prevalence rather than the possibility of entire prevention of intravenous drug abusers. Tattooing is also common in prisons and associated with increased risk of infection due to contaminated dyes and insufficient sterilized equipment being used. In Gilan, HCV infection was significantly associated with intravenous drug abuse, tattooing, and years of imprisonment. An overall decline of positive HCV carriers from 14.4% in 1999 to 4.5% in 2006 was visible (46).

### 2.8. Healthcare Preventive Strategies

Rigorous standard precautions in the public health system may also play an important role in transmission, which are not always taken seriously in developing countries. Reusing needles and syringes, and insufficiently sterilized medical instruments are also common in Egypt, which shows the highest percentage of new infections worldwide. The risk of HCV transmission due to sexual contact is possible especially for those with multiple partners. It was also reported that HIV infection increases the risk for an infection due to a weakened immune system (47). The risk of vertical transmission from infected mothers to their children is less than 5%. Although an improvement in the Iranian health system concerning endemic infectious diseases led to a decrease of HCV infection prevalence, there is a need to educational works to fill knowledge gaps about this disease; in particular, new methods have to be implemented to prevent transmission of this infection among prisoners.

### 2.9. Hepatitis D

Hepatitis D virus (HDV) is a serious infection that spreads worldwide and co-affects patients suffering from HBV. HDV requires HBV co-infection for its replication (48). HDV consists of three components of which one component actually originates from HBV (2). The spread of HDV follows that of HBV, particularly through parenteral exposure and secondly, by sexual transmission (2). HDV can either be acquired contemporary with HBV or superimposed later on a chronic hepatitis B. Studies reported a worldwide infection rate of HDV at approximately 5% of positive HBs Ag carriers (49). In regions where HBV is endemic, as in southern Europe or the Middle East, up to 50% of infected individuals also suffer from HDV. The clinical course of HDV depends on whether it is acquired simultaneously with HBV or a superimposed infection. When the infections happen simultaneously, HBV and HDV clearance is more likely resulting in a self-limiting course. HDV as a superimposed infection often leads to a more severe acute hepatitis. 

## 3. Results

As HDV never occurs without HBV infection, the immune responses to either of the two viruses cannot clearly be separated, making it difficult to investigate the exact pathogenesis exclusively for hepatitis D (48).

### 3.1. Hepatitis D in Iran

The Middle East including Iran is classified as endemic for HDV infection (50). In Iran, both effective HBV vaccination programs and enlightenment about transmission and prevention of infections were introduced successfully. In 1989, a study from Abbas *et al*. determined the presence of HDV in chronic HBV carriers with a prevalence rate of 14% in southern Iran (51). According to Taghavi et al. the rate declined drastically reaching to 9.7% from 2003 to 2004. However, completely different rates were reported from mid-west areas of Iran showing low prevalence rate of 2.4% in 1989 (46). Between 1986 and 1988, data were elicited concerning HDV expression in different high-risk groups investigating the clinical impact of hepatitis infection on chronic liver disease and HCC. Anti-HDV positivity was observed in asymptomatic HBsAg chronic carriers, hemophiliacs, and anti-HBsAg positive dialysis patients at 2.5%, 33.3%, and 44.5% of cases, respectively. Additionally, in 62.5% of patients affected by HCC the presence of hepatitis D virus infection was approved. Recent studies reported new data of prevalence rates of positive HDV among chronic HBV patients in different provinces (Table 4). In Tehran HDV prevalence was 5.7% in HBsAg carriers in 2004 (50). The reported prevalence in 2000 for the province of Golestan was 5.8% (52); for Kerman, 20.7%; and for Tabriz, 6.2%. In 2008, a study performed in the province of Kermanshah showed a high HDV prevalence of 31.6% in HIV/HBV co-infected patients (Table 3) (49).

**Table 3:** HDV infection prevalence in different Iranian provinces in the general Iranian population.

Province	Country region	Percentage of HDV Infection Prevalence	Year of Published Data
**Tehran**	Central	5.7	2004
**Kerman**	South-East	20.7	2003
**Golestan**	North	5.8	2007
**E. Azarbaijan (Tabriz)**	North-West	6.2	2000
**Mazandaran (Babol)**	North	2.0	2002
**Kermanshah ^a^**	West	31.6	2008

^a^HIV/HBV co-infected individuals.

All others: chronic HBV patients.

**Table 4:** The Incidence of Liver Cell Carcinoma in Various Countries, Expressed in Number of Cases/100.000 Inhabitants/Year

High Risk (20-150)	Intermediate Risk (5-20)	Low Risk(< 5)
**East, West and central Africa (Black population)**	South-East Asia and South Africa (Indians)	North, West and Central Europe
**South-East China**	Japan	North and South America
**Taiwan**	Middle East	Australia
**Korea**	India, Pakistan	North and South Africa (Arabs, White populations)
**Thailand**	South and East Europe	Central Asia
**Vietnam**	Central America	
**Burma**	Alaska	
**Hong Kong**		
**Singapore**		

### 3.2. Hepatocellular Carcinoma

HCC is a tumor with remarkable geographic differences about its incidences. Racial and genetic factors were shown to be irrelevant as risk factors for HCC, whilst environmental parameters such as hepatitis B and C infections and exposure to aflatoxin are closely related to HCC development (2) Countries can be distinguished into those with high-, intermediate- and low-risk regarding HCC incidence (Table 1). Especially in high risk countries, it must be considered that data collected by cancer registries are probably incomplete and therefore incidences are underestimated (2). In these countries, HCC is the commonest or next commonest neoplasm among all tumors (2) It is rather the maintenance of the lifestyle from the home countries that keeps environmental risk factors small in whites (2) The large variation in the incidence rate of HCC can be linked to the prevalence rates of HBV and, to a lesser extent, of HCV (2) Smoking, consumption of alcohol, and exposure to aflatoxins are risk factors for HCC (53). In all parts of the world, male gender, age, and liver cirrhosis are risk factors for HCC development. The mean age of patients in high-risk countries is lower compared to intermediate- and low risk areas (2). HCC evolves in a multi-step manner, so several causes sum up to HCC carcinogenesis (54). It is evident that the incidence increases with age in all populations, but in the elderly, the incidence falls off again. Interestingly, the age peak comes earlier in high-risk countries and patients are the oldest in low-risk countries. HCC also occurs in childhood, as mainly seen in high-incidence areas where children acquire hepatitis B infection early in life (2). The male-to-female distribution ranges between 4:1 in low-risk countries and 8:1 in high-risk countries. HCC may sometimes occur hereditary and seems to be more aggressive (55). In countries with poor hygienic standards, food is often spoiled with the carcinogenic mycotoxin Aspergillus flavus, and production of aflatoxins may occur (2). Other factors that contribute to HCC formation are oral contraceptives, androgenic/anabolic steroids, alcohol and tobacco (2). HBV and HCV infections are associated with higher risks for HCC (2). Geographical distribution of incidence for HCC and HBV infection indicates a closer link between the infection and the tumor (2) Some important points of evidence for etiological association between HBV and HCC include the greater risk of malignancy in cirrhosis due to HBV, presence of integrated HBV-DNA in HCC, and production of HBV antigens by HCC cell lines in the culture media (2). In both, HBsAg positive and negative patients the integration of hepatitis B virus promotes HCC development (2). Development of HCC is more likely when HBV infection occurs early in life, particularly at or near birth (53). The role of HDV in HCC development is difficult to evaluate since it never occurs without hepatitis B infection. Recent evidence indicates that HDV infection is harmful to the liver in combination with HBV (2). Patients with both HBV and HDV infections are more likely to develop cirrhosis and HCC at younger age compared to patients that suffer from HBV alone (56). In Taiwan an immunization program was initiated in 1984 which leads to halved HCC incidence (57) HCV has become an important risk factor for HCC, especially in intermediate-incidence areas such as the Middle East and the Mediterranean areas (58). The strongest association between HCV and HCC has been found in Japan, where post-transfusion hepatitis C in the 1950s and 1960s doubled HCC incidences over the last decades (58). Since HCV is integrated into the liver cell genome as HBV does, its carcinogenic effect is probably due to a necro-inflammatory process and development of cirrhosis (2)

### 3.3. Mouse Models for Viral Hepatitis Induced HCC

Based on the above-mentioned information on virus-associated hepatitis and its concomitant effects with progression to HCC, it seems helpful to examine this disease by using animal models. Until today, multiple rodent models were established highlighting the link between chronic inflammation, fibrosis, and liver cancer, as well as between virus-associated hepatitis and HCC (59). In this review some relevant animal models for virus-related hepatitis are discussed.

### 3.4. The HBV Large Envelope Protein Containing Alb-PSX Transgenic Mice

In a study by Chisari three transgenic mouse lines which express the HBV BbIII-A fragment under the transcriptional control of the albumin promoter were generated. The lines 45-2 (Tg[Alb-HBV]Bri43), 45-3 (Tg[Alb-HBV]Bri141) and 50-4 (Tg[Alb-HBV]Bri44) are immunologically inert to the HBV protein. Acute liver failure in this transgenic mouse model is caused by accumulation of the HBsAg inside the hepatic endoplasmic reticulum (ER) which is mediated via overexpression of the HBV large envelope protein. All three lines revealed significantly different amounts of hepatic HBV envelope proteins. In lineage 50-4 100% HBsAg were expressed in hepatocytes accompanied by strongly elevated expression level of the large and major HBV envelope protein. The expression of hepatic large and major HBV envelope polypeptides resulted in building long branching HBsAg filaments which accumulates in the ER and induces apoptosis of hepatocytes. Only the mouse lines 45-2 and 50-4 revealed significant liver failure reflected by enhanced serum glutamic-pyruvic transaminase (SGPT) levels. Examination of 2 to 3 month-old 50-4 mice revealed liver injury and after six months microscopic nodules were observed. Moreover, the expression pattern of hepatic HBsAg changed over time. After 9 to 12 months, mice of lines 45-2 and 50-4 displayed increased alpha fetoprotein (AFP) levels, and tumor formation was recognized. At the age of 18 months, all 50-4 mice with chronic liver disease showed hepatocellular neoplasms. Histological and cytological analyses of these tumors revealed a heterogeneous pattern while all could be classified as hepatocellular adenomas or HCCs. Occurrence and the certain histological features of the examined tumors in the 50-4 mice were affected by age, gender, and genetic factors. Male mice developed significantly more HCCs (72% males vs. 31% females) than adenomas compared to females that predominantly developed adenomas. These mice demonstrated that overexpression of components of the HBV virus proteins are able to promote chronic hepatitis followed by HCC development in a gender and genetic background dependent manner.

### 3.5. The TgAlb-1/HBV Mouse Model

It is commonly accepted that antiviral T cells have a major impact on regulating HBV-induced chronic hepatitis (60). It has been shown that all examined HCCs originate from long lasting chronic hepatitis (60). Alternatively, the enhanced copy numbers of HBV in infected hepatocytes and tumor cells may promote HCC progression. Impede HBV X gene expression, caused by the subviral DNA fragments, seems to be involved in HCC progression via cellular growth control (61). Studies by Chisari and colleagues revealed genetic modification of the HBV large envelope polypeptide expression level in mice as inducing factor for liver cell damage, compensatory proliferation, oxidative stress, glutathione depletion, transcriptional deregulation, and aneuploidy (62). To examine if a chronic and virus-specific immune response is able to mediate HCC development, Nakamoto and colleagues used a HBV-specific mouse model (63). The hepatocytes of the Tg (Alb-1, HBV) Bri66 mice showed non-toxic expression levels of small, middle, and large HBV envelop proteins and were immunologically inert against HBsAg with respect to T cell counts (63). 8 to 10 week-old transgenic male mice were thymectomized, irradiated, and divided into two groups for bone marrow reconstitution (BMR) with non-transgenic (group 1) or transgenic (group 2) T cell depleted donor bone marrow (60). Splenocytes infected with recombined vaccinia virus (HBs-vac, which expresses HBsAg) were injected in group 1 mice (composed of mice 1 week after thymectomy and irradiation) whereas in group 2 control animals were injected with splenocytes from immunologically tolerant transgenic littermates. Analyses were performed 3 weeks, 3 months, 8 months, and 17 months after bone marrow reconstitution with HBs-vac primed cells. Group 1 mice showed signs of hepatitis 3 weeks after infection (60). Seventeen months later all animals developed liver tumors with typical characteristics of HCC (60).

### 3.6. The TgAlb-1/HBV Mouse Model

It is generally assumed that abnormal expression of cytotoxic cytokines is one of the driving forces in hepatitis-induced HCC progression (64). The proinflammatory cytokines lymphotoxin (LT) α and LTβ belonging to the tumor necrosis factor (TNF) superfamily are generated by activated T-, B-, NK-, and lymphoid tissue inducer cells (65). Different studies demonstrated that the HCV core protein is able to induce the LTβR and TNFR1 signaling cascade under participation of the canonical or non-canonical NF-κB signaling cascade (66) which contribiutes to HCC formation. Haybaeck and colleagues used a transgenic mouse model with a hepatocyte specific LTα and LTβ expression. Two lines were primarily generated, one with low (tg1222) and one with high LT expression levels (tg1223), to examine the impact of the LTβR signalaing pathway on chronic hepatitis and hepatitis-induced HCC (67) At the age of 4 to 6 months, the livers of tg1223 mice showed signs of massive portal and lobular inflammation whereas tg1222 livers showed only mild portal inflammation (67). At the age of 12 months about 35% of tg1223 mice developed HCC. It was shown that only tg1223/Tnfr1^-/-^ mice developed a phenotype, which is closely related to that of tg1223. Nine months after birth, tg1223/Rag1^-/-^ and tg1223/ Ikkβ^∆hep^ livers showed no signs of hepatitis, increased hepatocyte, or oval-cell proliferation. Even in 18 month-old livers of tg1223/Rag1^-/-^ and tg1223/ Ikkβ^∆hep^ animals, no evidence of hepatitis and HCC formation was found. Additionally, pharmaceutical long-term blockage of the LTβR, using LTβR-Ig, led to significantly decreased chronic hepatitis rates in tg1223 mice. This mouse model demonstrated that LTβR plays a pivotal role in hepatitis-induced HCC onset and progression, and that disruption of LTβR signaling pathway may have the potential for clinical investigation.

## 4. Conclusion

The mouse models listed above offer a good insight into the development of HCC. As the understanding of the underlying molecular mechanisms are the basis to find out new treatment modalities and also preventive strategies, mouse models are essential pre-clinical tools for bringing forth new information. Treatment of chronic viral hepatitis and HCC is still a challenge, and in the future, targeted and more individualized therapy shall improve the outcome.
